# Distracted While Reading? Changing to a Hard-to-Read Font Shields against the Effects of Environmental Noise and Speech on Text Memory

**DOI:** 10.3389/fpsyg.2016.01196

**Published:** 2016-08-09

**Authors:** Niklas Halin

**Affiliations:** Department of Building, Energy and Environmental Engineering, University of GävleGävle, Sweden

**Keywords:** text memory, distraction, background speech, aircraft noise, road traffic noise

## Abstract

The purpose of this study was to investigate the distractive effects of background speech, aircraft noise and road traffic noise on text memory and particularly to examine if displaying the texts in a hard-to-read font can shield against the detrimental effects of these types of background sounds. This issue was addressed in an experiment where 56 students read shorter texts about different classes of fictitious creatures (i.e., animals, fishes, birds, and dinosaurs) against a background of the aforementioned background sounds respectively and silence. For half of the participants the texts were displayed in an easy-to-read font (i.e., Times New Roman) and for the other half in a hard-to-read font (i.e., Haettenschweiler). The dependent measure was the proportion correct answers on the multiple-choice tests that followed each sound condition. Participants’ performance in the easy-to-read font condition was significantly impaired by all three background sound conditions compared to silence. In contrast, there were no effects of the three background sound conditions compared to silence in the hard-to-read font condition. These results suggest that an increase in task demand—by displaying the text in a hard-to-read font—shields against various types of distracting background sounds by promoting a more steadfast locus-of-attention and by reducing the processing of background sound.

## Introduction

People working in indoor environments (e.g., schools and offices) are often exposed to distracting sounds deriving both from within (e.g., background speech) and outside the building (e.g., aircraft noise or road traffic noise), which can be problematic as background sound generally has a negative impact on cognitive performance (Szalma and Hancock, 2011; Klatte et al., 2013). More specific, background speech has proven to be detrimental to performance on several office-related tasks including text memory (Banbury and Berry, 1997; Bell et al., 2008), reading comprehension (Martin et al., 1988; Oswald et al., 2000; Sörqvist et al., 2010), proofreading (Jones et al., 1990; Venetjoki et al., 2006; Smith-Jackson and Klein, 2009), and writing (Sörqvist et al., 2012a). Moreover, environmental noise originating from aircrafts and road traffic has the potential to impair reading proficiency in children (e.g., Hygge et al., 2002; Hygge, 2003; Boman, 2004; Clark et al., 2013), as well as impairing text memory and attentional functions in adults (Hygge et al., 2003; Enmarker, 2004; Sörqvist, 2010; Schlittmeier et al., 2015). Thus, it is important to investigate simple solutions that can aid individuals to resist distraction from various types of sound. This study will use a reading task as a tool to test if increased task demand—by changing the font of the text to one that is harder to read—can shield against the detrimental effects of the aforementioned types of background sounds on text memory.

According to the duplex-mechanism account of auditory distraction there are at least two distinct ways in which sound can disrupt task performance (Hughes, 2014). One way is sound that interferes with the deliberate processing of the focal material (i.e., interference-by-process), and the other way is sound that diverts focus away from the focal task (i.e., attentional capture). From the interference-by-process view text memory is impaired by background speech because both the focal material (i.e., the text that is read) and background speech contain semantic information and therefore engage similar processes in the brain, which in turn harms task performance (Marsh et al.; 2008; Marsh and Jones, 2011). As aircraft noise and road traffic noise do not contain semantic information the effect of these types of sound on text memory might instead be explained by the sounds’ acoustical characteristics (e.g., frequency modulation, salience, and predictability) that potentiate attentional capture (Sörqvist, 2010; Klatte et al., 2013). However, it seems to be possible to overcome distracting sound by inducing a higher degree of concentration on the focal task (Sörqvist and Marsh, 2015). Of particular interest to the current study, are experiments that have shown that an increase in task demand—e.g., by making the to-be-remembered items harder to perceive (i.e., sensory load), by increasing the amount of information that have to be kept in memory (i.e., working memory load), or by loading the visual field with information (i.e., perceptual load)—protects against attentional capture (SanMiguel et al., 2008; Hughes et al., 2013), attenuates semantic auditory distraction of free recall (Marsh et al., 2015), shield visual-verbal task performance against background speech (Halin et al., 2014a,b), reduces the neural processing of background tones (Sörqvist et al., 2012b), and reduces the awareness of a novel tone (Macdonald and Lavie, 2011).

In two recent experiments, participants undertook either a proofreading task (Halin et al., 2014a) or a prose memory task (Halin et al., 2014b) against a background of silence or speech. In these two studies, task demand was manipulated by displaying the texts in different fonts; one that was an easy-to-read font (i.e., Times New Roman) and one that was a hard-to-read font (i.e., Haettenschweiler). Both experiments revealed an interaction between background sound condition and font type, such as detection of semantic/contextual errors in the proofreading task and recall on the prose memory task was impaired by background speech, but only when the texts were displayed in the easy-to-read font. In contrast, there was no effect of background speech when the texts where displayed in the hard-to-read font. Also, participants scored significantly higher on the prose memory task in the presence of background speech when the text was displayed in a hard-to-read font compared to an easy-to-read font. Hence, by forcing participants to reach a higher degree of attentional engagement in the focal task (i.e., concentrate harder), by manipulating the readability of the text, task performance was shielded against distraction (Linnell and Caparos, 2013; Sörqvist and Marsh, 2015). Arguably, the shielding effect arises because higher attentional engagement in the focal task leads to a more steadfast locus-of-attention (Hughes et al., 2013) and to reduced processing of background sound (Sörqvist et al., 2012b; Halin et al., 2015; Marsh et al., 2015).

The shielding effect that increased task demand seems to have on distractibility (Hughes et al., 2013) can thus be a way to overcome distraction from background sound. However, it is still unclear if this type of technique also would shield against the effects of environmental noise on text memory. Therefore, participants in the current study completed a reading task in the presence of four background sound conditions (i.e., silence, background speech, aircraft noise, and road traffic noise). Half of the participants read texts displayed in an easy-to-read font (i.e., Times New Roman), and the other half read texts displayed in a hard-to-read font (i.e., Haettenschweiler). These two fonts were chosen based on previous experiments whereby the Haettenschweiler font have been judged to be more demanding and more difficult to read compared to the Times New Roman font (Halin et al., 2014a,b). It was expected that background speech, aircraft noise and road traffic noise would impair text memory compared to silence, but only for participants in the easy-to-read font condition. There would be no effect of background sound condition in the hard-to-read font condition (i.e., an interaction between the background sound condition and the font condition). Based on the findings in Halin et al. (2014b) it was also expected that participants in the hard-to-read font condition would score higher on the memory test in the background speech condition compared to participants in the easy-to-read font condition. In addition, participants answered questions on how tired, mentally exhausted and concentrated they were before and after the experimental session. This was asked to see if, the supposedly benefit of, a hard-to-read font would come with a cost, insofar that participants in the hard-to-read font condition would feel more fatigued after the experiment compared to participants in the easy-to-read font condition. To summarize, this study aimed to (a) replicate that increased task demand (e.g., by displaying the text in a hard-to-read font) shields against the detrimental effects of background speech on text memory, (b) to investigate if an hard-to-read font also would shield against the effects of environmental noise on text memory, and (c) examining the effect that increased task demand (by changing the font of the text) has on self-reported fatigue (i.e., tiredness, mental fatigue, and concentration).

## Materials and Methods

### Participants

Fifty-six (29 women) Swedish students (mean age = 24.45 years, *SD* = 4.97) participated for a small honorarium. All reported normal hearing, normal or corrected-to-normal vision and Swedish as their native language. The study was conducted in accordance with the declaration of Helsinki and the ethical guidelines given by the American Psychological Association. All participants were adults and participated on informed consent. The experiment caused no harm to any party, no information that can be associated with individual participants has been made available to an external part, and no conflict of interest can be identified. Prior the experiment participants were told that the study investigated the effects of background sound on memory. Afterward participants were orally debriefed about the purpose of the study.

### Materials

#### Background Sound

All verbal material in this experiment was in Swedish. The background speech comprised a recording of a conversation between a female speaker and a male speaker taking turns talking about mundane topics (e.g., recreational activities). The speech sound file was 5 min long and was adjusted to real speech level at 60 dB(A) L_eq_ based on the recommendation of the measurement level method (i.e., all the silent parts of the speech signal is removed prior to calculating sound pressure level; IEC 60268-16, 2011). The aircraft noise consisted of a recording of an airborne airplane passing by. The passage took approximately 1 min and was repeated five times to create the 5 min long sound file. The road traffic noise consisted of a recording of a road crossing with varying traffic. The sound file was 75 s long and was repeated four times to create a 5 min long sound file. The sound pressure level of the aircraft noise and the road traffic noise were both adjusted to 60 dB(A) L_eq_. All sound files were adjusted by using the software Matlab.

#### Text Memory

**FIGURE 1:**
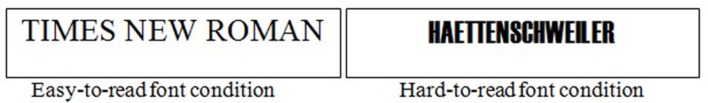
**Illustration on how the text was displayed in the easy-to-read font and hard-to-read font conditions**.

Four memory tests were developed and all tests had a reading phase and a test phase. In each reading phase participants read four paragraphs (∼ 130 words each) about fictitious species (one species per paragraph). The four fictitious species described in a memory test belonged to the same class of creatures and there were different classes used in the four memory tests (i.e., animals, fishes, birds, and dinosaurs). Each paragraph stated information about specific features (e.g., “The Malang have a 17–22 cm long tail”), habits (e.g., “The Malang is active during the day”) and habitats of a species (e.g., These animals [Malang] mainly live in large bushes). The paragraphs were either displayed in the font Times New Roman (i.e., easy-to-read font condition) or the font Haettenschweiler (i.e., hard-to-read font condition; see **Figure 1**). All paragraphs were written with 12-point font size, the spacing between lines set to 1, and with the left and right margins evenly adjusted. A paragraph was displayed for 75 s before it was automatically replaced by a new paragraph. After the time was up for the last paragraph participants were requested to answer if they had read all four paragraphs. Thereafter the computer went on to the test phase. In each test phase participants were asked to answer 24 multiple-choice questions that had the same 5 fixed options for all questions depending on which class of creature they had read about [e.g., all the questions on animals had these options: (A) Undon (B) Bonasus (C) Malang (D) Khian (E) None of the animals]. Participants were asked to select which of the five options was the correct answer to a question (e.g., Which animal has a 20 cm long tail). On 16 of the 24 questions options A to D were the correct answer (i.e., four correct answers for each of the options). The remaining eight questions had option E (i.e., None of the species) as the correct answer. On these questions a crucial piece of information was replaced with erroneous information: e.g., Which of the animals live in large trees? (i.e., in the text it says that the Malang lives in bushes). Each of the four species had two of these questions allocated to them. All questions were written in the Arial font and presented sequentially in the same fixed pseudo-randomly order for all participants. Each question was presented for 15 s before it was automatically replaced with the next question.

### Procedure and Design

A within-between mixed experimental design was used with font as the between-participant factor (easy-to-read font vs. hard-to-read font) and background sound as the within-participant factor (silence vs. background speech vs. aircraft noise vs. road traffic noise). At arrival, participants were randomly assigned to either of the two font conditions. Participants sat alone in a room in front of a computer screen. All instructions were presented on the computer and participants were instructed throughout the experiment to wear headphones and to ignore any sound. Next, participants answered a number of background questions (e.g., age and gender) and a short questionnaire of how tired, mentally exhausted and concentrated they were at the moment, by filling in a number between 1 to 9 that they felt best corresponded to their state of mind (e.g., 1 = not tired at all, 9 = very tired). Thereafter they undertook the four memory tests that were presented in the same fixed order for all participants [i.e., (1) animals, (2) fishes, (3) birds, and (4) dinosaurs]. The presentation order of the four background sound conditions were counterbalanced between participants in the same way for both font conditions.

## Result

### Text Memory

**FIGURE 2:**
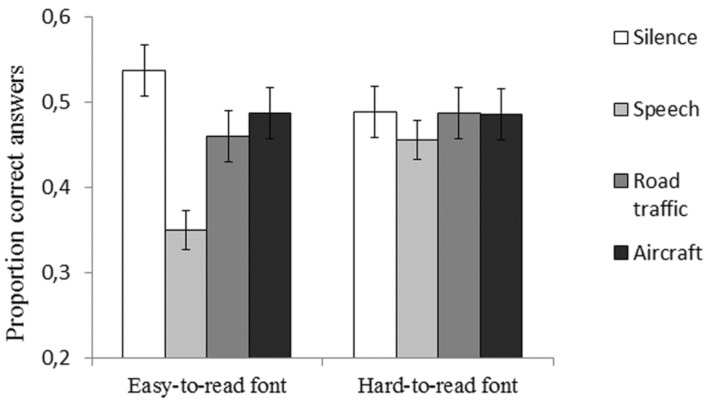
**Participants’ score on the reading task as the proportion of questions correctly answered across the four background conditions and the two font groups respectively.** Error bar represents standard error of means.

Only participants that followed task instructions and reported that they had read all paragraphs in the reading phase of the memory test were included in the analysis. As can been seen in **Figure 2**, performance on the memory test was impaired by background sound, but only for participants in the easy-to-read font condition, not for participants in the hard-to-read font condition. This conclusion was supported by a mixed 2 (Font condition: easy-to-read font vs. hard-to-read font) × 4 (Background sound condition: Silence vs. background speech vs. road traffic noise vs. aircraft noise) analysis of variance that revealed no main effect of font condition, *F*(1,54) = 0.49, *p* = 0.489, $\eta_{p}^{2}$ = 0.009, but a significant main effect of background sound condition, *F*(3,162) = 9.36, *p* < 0.001, $\eta_{p}^{2}$ = 0.15, and a significant interaction between the two factors, *F*(3,136) = 4.46, *p* = 0.005, $\eta_{p}^{2}$ = 0.08. In the easy-to-read font condition, planned contrasts (simple first) revealed that recall was significantly impaired by background speech, *F*(1,27) = 36.02, *p* < 0.001, $\eta_{p}^{2}$ = 0.57, road-traffic noise, *F*(1,27) = 9.07, *p* = 0.006, $\eta_{p}^{2}$ = 0.25, and aircraft noise, *F*(1,27) = 4.28, *p* = 0.048, $\eta_{p}^{2}$ = 0.14, compared to silence. In the hard-to-read font condition, there was no effect of background speech, *F*(1,27) = 1.16, *p* = 0.291, $\eta_{p}^{2}$ = 0.04, road-traffic noise, *F*(1,27) = 0.02, *p* = 0.966, $\eta_{p}^{2}$ < 0.01, or aircraft noise on text memory, *F*(1,27) = 0.13, *p* = 0.910, $\eta_{p}^{2}$ < 0.01, compared to silence. Moreover, participants in the hard-to-read font condition scored significantly higher on the memory test in the background speech condition compared to participants in the easy-to-read font condition [mean difference: 0.11, *t*(54) = 3.26, *p* = 0.002, *r* = 0.40]. No other comparisons between the two font conditions were significant (silence: *p* = 0.256, road-traffic noise: *p* = 0.530, aircraft noise: *p* = 0.971).

### Self-Reported Fatigue

Mean values on self-reported fatigue are presented in **Table 1**. Participants in the two font conditions did not differ from each other in how tired, mentally exhausted and concentrated they were prior to the experimental session. But, participants in both font conditions reported being more tired, more mentally exhausted and less concentrated afterward compared to how they felt before they started the experiment. A mixed 2 (Font condition: Easy-to-read font vs. hard-to-read font) × 2 (Time: Before session vs. after session) multivariate analysis of variance on tiredness, mental exhaustion and concentration revealed no significant main effect of font condition, *F*(3,52) = 0.23, Λ = 0.99, *p* = 0.877, $\eta_{p}^{2}$ = 0.01, but a significant main effect of time *F*(3,52) = 15.36, Λ = 0.53, *p* < 0.001, $\eta_{p}^{2}$ = 0.47, and no interaction between the two factors, *F*(3,52) = 0.32, Λ = 0.98, *p* = 0.809, $\eta_{p}^{2}$ = 0.02. The univariate test revealed that participants in both font conditions reported being more tired, *F*(1,54) = 28.86, *p* < 0.001, $\eta_{p}^{2}$ = 0.35, more mentally exhausted, *F*(1,54) = 27.13, *p* < 0.001, $\eta_{p}^{2}$ = 0.33, and less concentrated after the experiment compared to prior the experiment, *F*(1,54) = 30.57, *p* < 0.001, $\eta_{p}^{2}$ = 0.36.

**Table 1:** Mean values (standard deviation) on the self-reported data of how tired, mentally exhausted and concentrated participants were before and after the experimental session across the two font conditions.

	Easy-to-read font condition				Hard-to-read font condition			
		
	Before session		After session		Before session		After session	
				
Subjective measures	*M*	*SD*	*M*	*SD*	*M*	*SD*	*M*	*SD*
Tiredness	4.07	1.74	5.43	1.97	4.46	2.01	5.54	2.17
Mental exhaustion	3.86	1.80	5.25	1.90	4.11	2.20	5.57	2.32
Concentration	6.25	2.03	4.18	1.52	5.86	1.98	4.11	1.81


## Discussion

The purpose of this study was to investigate if an increase in task demand could shield against the detrimental effects of background speech, aircraft noise, and road traffic noise on text memory. As hypothesized, text memory was impaired by all three background sound conditions compared to silence, but only in the easy-to-read font condition not in the hard-to-read font condition. Also, participants in the hard-to-read font condition performed significantly better on the text memory test in the background speech condition compared to participants in the easy-to-read font condition. Moreover, the benefit of the hard-to-read font did not come with an additional cost insofar that participants in the two font conditions did not differ from each other in the magnitude of self-reported tiredness, mental exhaustion and lack of concentration before and after the experimental session.

The results that a hard-to-read font shields against distraction from background speech on text memory, and that memory performance in the background speech condition was significantly better for participants in the hard-to-read font condition compared to the easy-to-read font condition, replicates the finding of Halin et al. (2014b). The result of the current study also extend the findings of a trade-off between higher task demand and auditory distraction (Macdonald and Lavie, 2011; Sörqvist et al., 2012b; Hughes et al., 2013; Halin et al., 2014a,b; Marsh et al., 2015) to concern environmental noise (i.e., aircraft noise and road traffic noise). Thus, the main contribution of this paper is that it shows that it is possible to overcome distraction from various types of background sound while reading by increasing task demand. A simple way to achieve increased task demand is by changing the font of the text to one that is harder to read. Arguably, a hard-to-read font forces the reader to concentrate more on the text (Sörqvist and Marsh, 2015). The increased (attentional) engagement on the focal task gives the reader a more steadfast locus-of-attention (Hughes et al., 2013), and it reduces the processing of background sound (Sörqvist et al., 2012b; Halin et al., 2015; Marsh et al., 2015) at the face of presentation of the distracting sound (Marsh et al., 2015), and consequently, the reader is less distracted by background sound.

A question that has engaged previous research is whether background speech is more detrimental to text memory than aircraft noise or road traffic noise (e.g., Hygge et al., 2003; Enmarker, 2004; Sörqvist, 2010; Sætrevik and Sörqvist, 2015). In this study the lowest text memory score was found in the background speech condition when the text was displayed in the easy-to-read font (see **Figure 2**). This finding is in line with the result of Sörqvist’s (2010) study, wherein background speech was more harmful to prose memory than aircraft noise. Previous studies that have compared the effects of background speech and road traffic noise have found that both sounds impaired cued prose recall compared to silence, but that neither of the two sounds were more distracting than the other (Hygge et al., 2003; Enmarker, 2004). Yet, when text memory was tested with recognition questions (i.e., multiple-choice questions) there was only an effect of background speech in Enmarker’s (2004) study, but there was no effect of sound in Hygge et al.’s (2003) study. In contrast, this study found an effect of both speech and environmental sound on text memory by using recognition questions. A major difference between this study and that of Hygge et al. (2003) and Enmarker (2004) was that they had composed the sounds to resemble each other’s acoustical properties, which this study did not do. Hence, any differences in the magnitude of how distracting each sound was could be due to how the sounds were altered. Still, based on the assumption made by the interference-by-process view it would be expected that background speech is more detrimental to text memory than environmental sound. This because of the clash that occurs between the processes involved in the reading task and the automatically processing of the background speech (Marsh et al., 2008). Moreover, fMRI data indicates that different cognitive processes are activated when performing an updating task in presence of either background speech or aircraft noise, and that speech impairs the cognitive control function involved in updating information in working memory (Sætrevik and Sörqvist, 2015). Given that updating is important to text processing (De Beni et al., 1998), the dissimilarity in how the sounds influences neural activity might explain why background speech was the most detrimental sound condition in the current experiment.

Even though background speech stands out as the most distracting sound source compared to silence, the result of the current study suggests that environmental noises are also harmful to text memory. From an applied point of view this finding is important to consider because it highlights that both speech and environmental noise are potential distracters in environments where it is crucial to be able to remember written information (e.g., schools and offices). However, the question is how applicable it is to increase task demand on a text (e.g., by changing the font to one that is harder to read) while reading in a noisy real-life setting? When people are reading at work or in school they can do so for a longer period of time than was the case in this experiment (i.e., 5 min per text). Also, in daily life people often must recall what they read far longer from encoding than what participants did in this experiment where text memory was measured directly after reading. Further research is needed to investigate the long-term consequences of reading a text displayed in a hard-to-read font. Regarding both the impact habituation has on the shielding effect of a hard-to-read font, and if increased task demand also has positive long-term effects on text memory. Another important issue is whether increased task demand would lead to higher levels of arousal that combined with a noisy environment would impose an increased health risk to people (Andringa and Lanser, 2013). The result in this study on self-reported tiredness, mental fatigue and concentration showed that the hard-to-read font was not perceived as more mentally exhausting than the easy-to-read font, at least in the short duration of time it took to complete the current experiment (∼20 min). However, this study did not investigate the effects of increased task demand on annoyance or irritability, which also are factors that could impose a health risk to people, e.g., by producing physical and psychological stress (Ouis, 2001; Andringa and Lanser, 2013). These matters are important to further investigate in order to answer if increased task demand is a sustainable way to overcome auditory distraction outside of the laboratory.

## Conclusion

This paper shows that a hard-to-read font, at least temporary, can boost concentration and shield against the detrimental effects of environmental noise and background speech on text memory. Hence, a simple alteration of the appearance of a text can help individuals that are reading in noisy environments to overcome auditory distraction.

## Author Contributions

The author confirms being the sole contributor of this work and approved it for publication.

## Conflict of Interest Statement

The author declares that the research was conducted in the absence of any commercial or financial relationships that could be construed as a potential conflict of interest.
